# Perception of tobacco hazards on general and periodontal health and tobacco cessation counseling among dental students

**DOI:** 10.18332/tpc/175951

**Published:** 2024-01-22

**Authors:** Jazia A. Alblowi

**Affiliations:** 1 Periodontology Department, Faculty of Dentistry, King Abdulaziz University, Jeddah, Saudi Arabia

**Keywords:** knowledge, smoking, attitude, belief, tobacco cessation counselling, dental students

## Abstract

**INTRODUCTION:**

Tobacco use is one of the leading worldwide health risk factors and a primary cause of premature death and disability. Tobacco cessation programs might work well if provided by all healthcare providers. This study aimed to evaluate dental students' knowledge, attitudes, beliefs, and practices towards tobacco hazards on general and periodontal health and tobacco cessation councling.

**METHODS:**

A descriptive cross-sectional study was conducted among dental students who were in their clinical years (the fourth, fifth and sixth year of study), in Saudia Arabia in 2022. A self-administered questionnaire derived from the TCC questionnaire survey was carried out among 315 dental students. Knowledge was considered poor if correct answers were below the median value. Attitude was on a five-point Likert scale. Adjusted logistic regression analyses were performed.

**RESULTS:**

The study revealed that about 52% have poor knowledge, 64% have negative attitudes, 69% have negative beliefs, and 44% poor practice. All these ratings were below median values. It also showed that younger dental students were 2 times more likely to have poor knowledge (AOR=1.97; 95% CI: 1.1–3.53) and smokers were less likely to have poor knowledge (AOR=0.34; 95% CI: 0.12–0.95). One third of students perceived patient resistance as a barrier to TCC while 50% reported lack of knowledge, 32% lack of time, and 24% lack of materials.

**CONCLUSIONS:**

The study findings urge the inclusion of programs to encourage dental students to help patients quit tobacco use and to make educational material available to them.

## INTRODUCTION

Tobacco use is a major global health hazard and a leading cause of early death and disability. It causes seven million deaths every year, as reported by the World Health Organization (WHO)^1^. Smoking leads to serious health problems, such as heart disease, lung disease, cancer, reproductive health issues, and increased vulnerability to infectious diseases^2^. The number of smokers in the developing world is growing rapidly, posing a grave threat to global health now and in the future. Unless the global rate of tobacco use changes, the world’s smoking population, which is currently around 1.3 billion, will rise to 1.7 billion by 2025^3^.

All healthcare delivery systems need to initiate and carry out tobacco cessation programs. Medical workers have traditionally been the main providers of many healthcare services, including tobacco cessation programs. These services can be improved by involving those who participate in healthcare delivery. Other healthcare providers, such as dentists and their staff, can also play a vital role in reducing tobacco use among the general population^4,5^. Dental practitioners, as members of a multi-professional healthcare team, are well suited to help prevent and support smoking cessation. As smoking produces hazardous effects on oral and periodontal health; periodontists in particular are entitled to face the harmful effects of smoking such as gingivitis, altered tissue response to tobacco noxious elements, attachment loss, and consequently tooth loss^6^. Meanwhile, future dental practitioners and dental students examine the oral cavity and often encounter medical emergencies in dental clinics^7^. They often see tobacco-related signs on oral tissues, such as tooth discoloration, bad breath, precancerous lesions, and cancerous lesions. They perform dental treatment which may require multiple visits, and therefore provide an opportunity to start, strengthen, and support tobacco cessation interventions^8,9^.

Many studies have demonstrated that even brief and simple advice from health professionals can significantly lower smoking rates, and that prevention and cessation counseling in the dental setting is both appropriate and effective^10-12^.

In Saudi Arabia health services, including dental services, are provided in the governmental part mainly by the Ministry of Health. At the primary healthcare level, health centers distributed in all sectors of the country with a referral level to general hospitals (secondary level) and to specialized hospitals at the tertiary level for all citizens and residents for free. The Ministry of Education provides free services for students and employees as well as to the population^13^.

Therefore, the current study was planned and conducted in the main governmental dental school in the second city of Saudi Arabia. Moreover, this dental faculty could represent faculties with a system and curriculum that is accredited by Academic recognition by the Association for Dental Education in Europe (ADEE) and American Accreditation (CODA), and the study aimed to explore the dental students’ knowledge, attitude and behavior towards tobacco hazards on general and periodontal health and tobacco cessation counseling as future members of the global dental community.

## METHODS

The research was a cross-sectional descriptive survey conducted between March and May 2022 among dental students in their clinical training years (4th, 5th, and 6th year) using a convenience sampling technique (n=315). All students of the three years were approached. The rationale of the study was explained to the participants, and they voluntarily took part in the study after giving verbal consent. No compensation or incentives were given to participating students and their participation was anonymous. The Ethics Committee of the Dental Faculty permitted this study to proceed under ethical consideration.

The questionnaire was prepared after a thorough review of literature on the topic and it was derived from a previous study^14^ and utilized the tobacco cessation counseling survey^14^ which was used in previous research among dental professionals in Saudi Arabia. It was tested by three experts in tobacco cessation to ensure its content validity and was pretested for face validity and clarity. The questionnaire covered participants’ demographic data, their smoking status, and assessed knowledge, attitude, and practice of tobacco cessation counseling. Questions on perceived barriers to practicing TCC were also included.

Knowledge questions were on four domains, concerned with the effects of smoking on general health, oral health, the prevalence of smoking in Saudi Arabia, and the various treatment modalities to quit smoking. Answers were classified as poor if they were less than the median value, and good if they were higher. Attitude questions sought the perception of professional responsibility towards smoking cessation advice, and if perceived as being within the scope of the dentist, their willingness to provide that advice and on their belief in its effectiveness. They were rated on a five-point Likert scale ranging from ‘strongly agree’ to ‘strongly disagree’. However, it was finally dichotomized into positive and negative attitudes. Practice questions inquired about having provided TCC or not, and the barriers to providing it. Logistic regression analysis was performed to examine the factors related to students’ knowledge, attitude, beliefs and behaviors on TCC. These variables were the dental study year, gender, and smoking status if they were current or previous smokers as well as family smoking habits.

SPSS version 21.0 software (Chicago, IL, USA) was used to conduct descriptive and chi-squared analyses on the data. A p<0.05 was considered statistically significant.

## RESULTS

The study included 315 students: 43.8% males and 56.2% females with a mean age of 23.03 ± 1.45 years. The response rate among the sample was 76.2%, while by year of study was 80.3%, 68.8% and 81.5% among males in 4th, 5th and 6th year, respectively. The corresponding response rates among females were 88.7%, 74.7%, and 85.3%, respectively, with no significant difference in response rate by gender or year of study. Current smokers constituted 23.8% of students, mainly cigarettes, and 28.9% of students reported family smoking. Students with less than median scores of knowledge, attitude, belief, or practice, were considered to have poor knowledge, negative attitude and poor practice. Results are given in Tables 1 and 2. Overall, about 52% had poor knowledge, 64% had negative attitude, 69% had negative beliefs, and 44% had poor practice. Poor knowledge was more prevalent among females (49.7% and 49.8%, respectively). Negative attitude was significantly more frequent among students in 4th year, and those with family smoking. Practices were poor among students in 6th year and current smokers. More details are given in the Supplementary file.

**Table 1:** Good^†^ knowledge (%) related to tobacco cessation counselling in association with dental students’ characteristics, cross-sectional descriptive survey, March to May 2022, Saudi Arabia (N=315)

*Characteristics*	*Knowledge*				
	*General effects of smoking*	*Oral and periodontal effect of smoking*	*Smoking in KSA*	*Treatment modalities*	*Overall*
**All**	46.7	44.8	22.9	33.7	44.8
**Year**					
4th	41.1	61.3	29.0	40.3	51.6
5th	51.7	30.3	20.2	24.7	38.2
6th	49.0	37.3	17.6	33.3	42.2
p	0.265	<0.001*	0.040*	0.059	0.124
**Gender**					
Male	43.5	31.2	17.4	34.8	38.4
Female	49.2	55.4	27.1	32.8	49.7
p	0.317	<0.001*	0.041*	0.707	0.045*
**Current smoker**					
No	50.6	44.5	26.7	36.8	49.8
Yes	32.4	45.6	8.8	22.1	26.5
p	0.008*	0.877	0.002*	0.022*	0.001*
**Family smoking**					
No	50.0	45.5	21.9	30.4	44.6
Yes	38.5	42.9	25.3	41.8	45.1
p	0.063	0.665	0.515	0.052	0.947

† Answers were classified as poor if they were less than the median value, and good if they were higher.

* p<0.05 (significant).

**Table 2:** Attitudes, beliefs and practices (%) related to tobacco cessation counselling in association with dental students’ characteristics, cross-sectional descriptive survey, March to May 2022, Saudi Arabia (N=315)

*Characteristics*	*Attitude*				*Belief in effectiveness of TCC*	*Practice*
	*Professional responsibility*	*TCC is within the scope of dentistry*	*Willingness to provide TCC*	*Overall*		
**All**	41.3	48.6	34.3	48.3	49.5	43.5
**Year**						
4th	49.2	52.4	54.8	63.7	69.4	44.4
5th	23.6	51.7	12.4	27.0	30.3	22.5
6th	47.1	41.2	28.4	48.0	42.2	60.8
p	<0.001*	0.191	<0.001*	<0.001*	<0.001*	<0.001*
**Gender**						
Male	41.3	44.2	27.5	44.2	31.2	42.0
Female	41.2	52.0	39.5	51.4	63.8	44.6
p	0.991	0.171	0.026*	0.204	<0.001*	0.644
**Current smoker**						
No	37.7	47.8	31.6	46.6	49.8	39.7
Yes	54.4	51.5	44.1	54.4	48.5	57.4
p	0.013*	0.589	0.054	0.251	0.853	0.009*
**Family smoking**						
No	40.2	48.2	29.0	44.6	49.1	40.6
Yes	44.0	49.5	47.3	57.1	50.5	50.5
p	0.537	0.842	0.002*	0.044	0.816	0.107

Answers were classified as poor if they were less than the median value, and good if they were higher. Attitude questions sought the perception of professional responsibility towards smoking cessation advice, and if perceived as being within the scope of the dentist, their willingness to provide that advice and on their belief in its effectiveness. They were rated on a five-point Likert scale ranging from strongly agree to strongly disagree. However, it was finally dichotomized into positive and negative attitudes. Practice questions inquired about having provided TCC or not and the barriers towards providing it.

* p<0.05 (significant).

About one-third of students perceived patient resistance as a great barrier to TCC. When students were asked about reasons for not including TCC in their practice, 50% reported a lack of enough knowledge, 32% lacked time, and 24% lack of necessary materials.

Logistic regression analysis revealed that students in 4th year were 2 times (AOR=1.97; 95% CI: 1.10–3.53) more likely to have poor knowledge compared to 5th year, and smokers were less likely to have poor knowledge (AOR=0.34; 95% CI: 0.12–0.95) (Table 3). Significant factors related to negative attitude included year, 4th and 6th years were more likely to have negative attitude compared to 5th year (OR=3.22; 95% CI: 1.58–6.55 and OR=2.63; 95% CI: 1.30–5.33, respectively) and smokers were 11 times (OR=10.6; 95% CI: 2.08–54.5) more likely to have negative attitude, poor knowledge and negative belief (poor knowledge and negative belief are significantly associated with negative attitude). Significant factors in relation to belief include year, students in 4th year were 7 times (OR=7.42; 95% CI: 3.39–16.2) more likely to have negative belief compared to those in 5th year, and poor knowledge and negative attitude (poor knowledge and negative attitude are significantly associated with negative belief). Considering student practices in relation to TCC, significant factors include gender, current smoking, and knowledge. Poor practices were more likely among students in 6th year (OR=5.88; 95% CI: 2.84–12.2), females, smokers, and those with poor knowledge.

**Table 3:** Logistic regression analysis of factors associated with dental students’ knowledge, attitude, belief and practices related to tobacco cessation counselling, cross-sectional descriptive survey, March to May 2022, Saudi Arabia (N=315)

*Variable*	*Knowledge*		*Attitude*		*Belief*		*Practice*	
	*AOR*	*95% CI*	*AOR*	*95% CI*	*AOR*	*95% CI*	*AOR*	*95% CI*
**Year**								
4th	1.97*	1.10–3.53	3.22*	1.58–6.55	7.42*	3.39–16.24	1.52	0.74–3.11
5th *®*	1		1		1		1	
6th	1.28	0.70 –2.33	2.63*	1.30 –5.33	1.95	0.94 –4.06	5.88*	2.84 –12.18
**Gender**								
Male	0.77	0.24 –2.50	1.34	0.69 –2.59	0.16*	0.08 –0.31	1.39	0.73 –2.65
Female *®*	1		1		1		1	
**Current smoker**								
No *®*	1		1		1		1	
Yes	0.34*	0.12 –0.95	10.64	2.08 –54.51	4.48	0.83 –24.29	15.58*	3.47 –69.84
**Gender Smoking**	0.93	0.25 –3.39	0.09*	0.02 –0.57	0.35	0.05 –2.39	0.10*	0.02 –0.55
**Family smoking**								
No *®*	1		1		1		1	
Yes	0.83	0.48–1.43	0.86	0.45–1.63	1.75	0.89–3.44	1.08	0.59–1.98

AOR: adjusted odds ratio. ® Reference categories.

* p<0.05 (significant). Logistic regression analysis was performed to examine the factors related to the students’ knowledge, attitude, beliefs and behaviors on TCC. These variables were the dental study year, gender, smoking status if they were current or previous smokers as well as family smoking habits.

A significant smoking × gender interaction was found with attitude and practices, so the analysis was repeated after splitting the file by gender. Female smokers were more likely to have negative attitudes and poor practices than males (OR= 3.38; 95% CI: 1.27–8.96) (Table 4). Males in the 4th year (OR=57.3; 95% CI: 6.67–491) and 6th year (OR=24.0; 95% CI: 2.67–215) were more likely to have negative attitudes compared to 5th year students, while females of the same two years were more likely to have poor practices. Males with family smokers were more likely to have negative attitudes, but not females.

**Table 4:** Logistic regression analysis of factors associated with students’ attitude and practices related to tobacco cessation counselling after stratification by gender, cross-sectional descriptive survey, March to May 2022, Saudi Arabia (N=315)

*Variable*	*Males*				*Females*			
	*Attitude*		*Practice*		*Attitude*		*Practice*	
	*OR*	*95% CI*	*OR*	*95% CI*	*OR*	*95% CI*	*OR*	*95% CI*
**Year**								
4th	57.27*	6.67–491	0.29	0.07–1.25	0.63	0.23–1.73	3.38*	1.27–8.96
5th *®*	1		1		1		1	
6th	23.96*	2.67–215	3.49	0.97–12.64	1.28	0.49–3.31	5.68*	2.06–15.67
**Current smoker**								
No *®*	1		1		1		1	
Yes	1.08	0.43–2.69	1.78	0.70–4.53	13.91*	2.35–82.37	6.77*	1.43–31.97
**Family smoking**								
No *®*	1		1		1		1	
Yes	1.20	0.41–3.46	3.90*	1.41–10.81	0.48	0.20–1.18	0.45	0.19–1.05

® Reference categories.

* p<0.05 (significant).

## DISCUSSION

Dentists can contribute to the prevention of systemic and oral diseases and encourage a healthier lifestyle for their patients through tobacco cessation counseling (TCC)^15^. By being aware of the harmful effects of smoking and the methods of TCC, dental professionals can guide their patients toward quitting smoking and avoiding its complications. Results of the present study showed that the overall response rate among the study sample was76.2%, which is similar to other studies such as Aljubran et al.^16^ and Maharani et al.^17^, with response rates of 77.7 and 78.45%, respectively. The study found that 23.8% of students were current smokers, primarily cigarette, which is less than the level reported in previous studies^14,18^. The reason can be attributed to the different age groups, since these two studies were conducted among dental professionals and not students. However, compared with the level of dental students’ smoking in other countries, such as the US, the current study rate of smoking among dental students is much higher, since in an American Dental school^4^ it was found that only one student in 4th year (out of 177) was a current smoker, a percentage of 4.5% were occasional smokers, and about 7.6% had a previous history of smoking. This could be very alarming due to the risk of having a higher number of dental students in Saudi Arabia practicing this habit while they are entitled to advise patients to stop. However, this result cannot be generalized to all dental Saudi students since the data are only obtained from one dental faculty, which can be considered as a limitation of the study.

The current study also revealed that half of the participants lacked adequate knowledge. On average, 52% had poor knowledge, 64% had negative attitudes, 69% had negative beliefs and 44% had poor practice. This low level of knowledge agrees with a study conducted in 2015 in Bangalore (India) which reported that only 19% of the dental professional participants recognized the preventive measures of tobacco replacement therapy. Moreover, the present study also revealed that females showed lower levels of knowledge than males, which may be explained by many factors such as their lack of access to educational material relating to TCC, fear of recognition as smoker females in a conservative community, which makes it more difficult for them to attain the proper cessation advice directed specially to females. Regarding attitude, students in 4th year and those who had family members who smoked had more negative attitudes. This seems to be related to the fourth year as the start of clinical training and contact with patients, therefore, those younger students still lack courage and communication with their patients, while those with family smokers probably derive a negative impression from those smoker relatives. Sixth-year students and current smokers had worse practices. The study also found that 23.8% were smokers themselves, which might have influenced their perception or reflected their own situation. Therefore, it is important to engage the dentists in a constructive dialogue about the value of their involvement in TCC^15^.

The study also assessed the undergraduate training, and practice of TCC among the participants. It found that only 7% had formal training on TCC. When asked why they did not include TCC in their practice, the main reasons were lack of knowledge (50%), lack of time (32%), and lack of materials (24%). Approximately the same results were reported by the author^14^, but among dental professionals of different levels and specialties, where patient resistance (49.9%), and insufficient time, knowledge or training (45–48%) were the main barriers to TCC among them. These barriers seem to exist in the educational as well as the health delivery system in Saudi Arabia, being reported first by practitioners and then also by students. The previous study consequently suggested that education and training on TCC should be implemented and integrated into dental practice.

Similar results also supporting the current study were reported by Davis et al.^19^, who explored the views and attitudes of the British Dental Association members on implementing tobacco cessation strategies in their practices. They identified the main obstacles to a successful tobacco cessation campaign as the time needed, the lack of payment, the lack of training, the lack of patient education materials, and the lack of knowledge of referral resources. These results relating to countries with adequate financial and other resources such as England and Saudi Arabia indicate that the gap of applying TCC exists in strategies that do not integrate it as an essential part of dental education and health policies.

The study used logistic regression analysis to examine the factors that affect the knowledge and attitude of dental students toward TCC. It found that fourth-year students had twice the odds of having poor knowledge than the fifth-year students, which was previously explained by their younger age and being at the start of clinical training and actual contact with patients. Moreover, the periodontology teaching and training, whereby they get the bulk of tobacco knowledge and prevention, is yet at its primary stage, while smokers had better knowledge than non-smokers. This may be related to their worries and anxiety about adopting this harmful health habit, therefore they acquire more knowledge about it. Again, it was also found that year of study (4th and 6th years had more negative attitudes than 5th year); relative to sixth-year students, they were expected to adopt a better attitude, but their being engaged in a heavy curriculum with a lot of competencies based clinical requirements could explain their negative attitude towards TCC. The study highlighted the important role of smoking status in shaping the attitudes and practices of TCC. As expected, non-smokers had more positive attitudes than smokers. This is consistent with many previous studies that reported a detrimental effect of smoking status on dentists’ readiness and attitudes towards TCC^15,18,20,21^. Therefore, tobacco use should be avoided by dental practitioners to set a good example for their professional and patient communities.

### Strengths and limitations

The strength of the current study is that it allowed free expression of knowledge and opinion of the students to be anonymous. Subsequently, it captured a gap of knowledge in the topic and some barriers for participation in patient’s counseling. It may be one of the very few studies conducted in Saudi Arabia to address the readiness of dental students to participate in TCC. However, as a self-administered questionnaire reporting bias is expected to occur; also as the participants are dental students, reporting of desirable answers might have occurred. Another limitation was that the study only included dental students from one institution, which might limit the applicability of the results to other dental students.

## CONCLUSIONS

The study findings provided an important insight into the dental students’ knowledge, attitudes, and practices towards their role in smoking cessation. It is important to highlight the great need to improve dental students’ awareness and preparedness. The dental school should promote the importance of dental students and future professionals in helping patients to quit tobacco use. Despite the fact that the TCC topic is part of the curriculum of most dental schools in the world, a teaching course of tobacco use cessation should be added into the curriculum, which should include knowledge-based lectures, problem-based learning, and clinical practices with follow-up strategies to ensure its inclusion as daily-based practice toward smoking patients. Therefore, studies including other dental settings are desirable and modifications in the dental curriculum to bridge the anticipated gap of knowledge are recommended, especially relating to oral and periodontal effects of smoking that can be reinforced in the periodontology course. These studies utilizing evidence-based knowledge on tobacco cessation and including the 5As approach of handling the smoking situation would help involve the students in this important health problem.

## Data Availability

The data used to support the findings of this study are available from the author upon request.
